# HIV retesting for pregnant and breastfeeding women across maternal child health services in Nampula, Mozambique

**DOI:** 10.1371/journal.pone.0283558

**Published:** 2023-03-24

**Authors:** Chloe A. Teasdale, Michelle Choy, Fatima Tsiouris, Eduarda Pimentel De Gusmao, Etelvino C. P. Banqueiro, Aleny Couto, Kwalila Tibana, Nicole Flowers, Marilena Urso, Mirriah Vitale, Elaine J. Abrams

**Affiliations:** 1 Department of Epidemiology and Biostatistics, CUNY Graduate School of Public Health and Health Policy, New York, NY, United States of America; 2 Department of Epidemiology, Mailman School of Public Health, Columbia University, New York, NY, United States of America; 3 ICAP at Columbia University, Mailman School of Public Health, Columbia University, New York, NY, United States of America; 4 Ministério da Saúde de Moçambique, Maputo, Mozambique; 5 US Centers for Disease Control and Prevention, Atlanta, GA, United States of America; 6 US Centers for Disease Control and Prevention, Maputo, Mozambique; 7 Department of Pediatrics, Vagelos College of Physicians and Surgeons, Columbia University, New York, NY, United States of America; Centre de Recherche en Cancerologie de Lyon, FRANCE

## Abstract

**Background:**

Repeat HIV testing during pregnancy and breastfeeding identifies women with incident infections, those living with HIV who have been lost to care, and infants at risk for HIV infection. We report data from repeat testing for women in maternal and child health (MCH) services at 10 health facilities in Mozambique.

**Methods:**

Routinely collected data from health facility registers are reported from April-November 2019. From antenatal care (ANC), we report numbers and proportions of women eligible for retesting; returned for care when retesting eligible; retested; and HIV-positive (HIV+) at retesting. From child welfare clinics (CWC), we report mothers retested; tested HIV-positive; HIV+ mothers linked to ART services; HIV-exposed infants (HEI) tested for HIV with polymerase chain reaction (PCR) tests; HEI testing PCR positive; PCR-positive infants linked to care.

**Results:**

In ANC, 28,233 pregnant women tested HIV-negative at first ANC visit, 40.7% had a follow-up visit when retesting eligible, among whom 84.8% were retested and 0.3%(N = 26) tested HIV+. In CWC, 26,503 women were tested; 0.8%(N = 212) tested HIV+ and 74.1%(N = 157) of HIV+ women were linked to care. Among 157 HEI identified in CWC, 68.4%(N = 145) received PCR testing and 19.3%(N = 28) tested positive.

**Conclusion:**

In ANC, less than half of pregnant women eligible for retesting returned for follow-up visits, and test positivity was low among women retested in ANC and CWC. In CWC, linkage to infant testing was poor and almost 20% of HEI were PCR-positive. Implementing retesting for pregnant and breastfeeding women is challenging due to high numbers of women and low testing yield.

## Background

Identifying pregnant women living with HIV (WLHIV) and initiating them on antiretroviral therapy (ART) is critical for maintaining their health and decreasing the risk of vertical transmission [1]. Expansion of HIV testing for all pregnant women at the first antenatal care (ANC) visit with immediate ART initiation has led to significant reductions in new pediatric HIV infections [2]. In 2010 across UNAID’s 21 focus countries (those with the largest populations of pregnant WLHIV), less than half of all pregnant WLHIV received ART and there were more than 250,000 new pediatric infections, whereas in 2020, 85% of pregnant women received ART and approximately 110,000 children newly acquired HIV [2]. Despite high coverage of HIV testing and ART among pregnant women there are still many children newly acquiring HIV each year [1, 3, 4].

There are several factors driving vertical transmission of HIV, including poor retention of WLHIV in care and on ART [5]. Loss to follow-up from prevention of mother-to-child transmission (PMTCT) and ART services during pregnancy and in the postnatal period increases the risk of vertical transmission and compromises women’s health [2]. It is estimated that almost a third of women in high HIV burden countries who initiate treatment during pregnancy are not retained by 6 months [5]. In addition, incident HIV infections occurring during pregnancy and in the postnatal period are contributing to an increasing proportion of new pediatric HIV infections [2, 6]. When women newly acquire infection during pregnancy or breastfeeding, there is higher risk of vertical transmission [7] and these women are often missed for interventions, including ART initiation, infant postnatal prophylaxis and early infant diagnosis (EID) [8].

The World Health Organization (WHO) recommends repeat HIV testing for pregnant and postpartum women in countries with generalized HIV epidemics to identify women with incident infections occurring during pregnancy and breastfeeding [9]. Repeat testing in ANC, postnatal and maternal child health (MCH) services, such as family planning and pediatric immunization clinics, are recommended. Repeat testing done in these settings can also be used to identify and reengage WLHIV who previously tested positive but were lost to care, as well as to engage women who did not deliver in health facilities [4]. While many countries have adopted national guidelines for retesting pregnant and breastfeeding women [10], there is limited information about how effectively these guidelines are being implemented in high HIV burden, low resource settings, including information about strains on the healthcare system from increased testing volume [11, 12]. A study of routinely collected data from Kenya, which has had repeat testing guidelines since 2012, found that only 27% of pregnant women who tested HIV-negative at the first ANC visit and returned for a follow-up visit when eligible for retesting (>12 weeks) received a test [13]. In Tanzania, where national guidelines also call for retesting, among women who had delivered in a health facility and had tested HIV-negative during pregnancy, only 30% were retested at the time of delivery; among those tested, 13.3% were found to be HIV-positive [14]. These data suggest significant challenges to implementation of repeat testing for pregnant and postnatal women and emphasize the critical role of retesting in detecting incident HIV infections.

UNAIDS data for Mozambique show that in 2020, most of the infections acquired by children during pregnancy were because mothers dropped off treatment, and among children acquiring infection during breastfeeding, more than 40% were the result of mothers with incident infections [2]. In order to support full implementation of national HIV testing guidelines for pregnant and breastfeeding women, ICAP at Columbia University, in collaboration with the Mozambican Ministry of Health (MOH) and with funding from the US Centers for Disease Control and Prevention (CDC), provided technical assistance at 10 health facilities in 2019 [15]. Using routinely collected data we report testing coverage and positivity from ANC services, test-positivity among postnatal women in MCH services, and test-positivity and referral for treatment among infants of mothers tested in child welfare clinics (CWCs).

## Methods

### Project implementation

**Fig 1:**
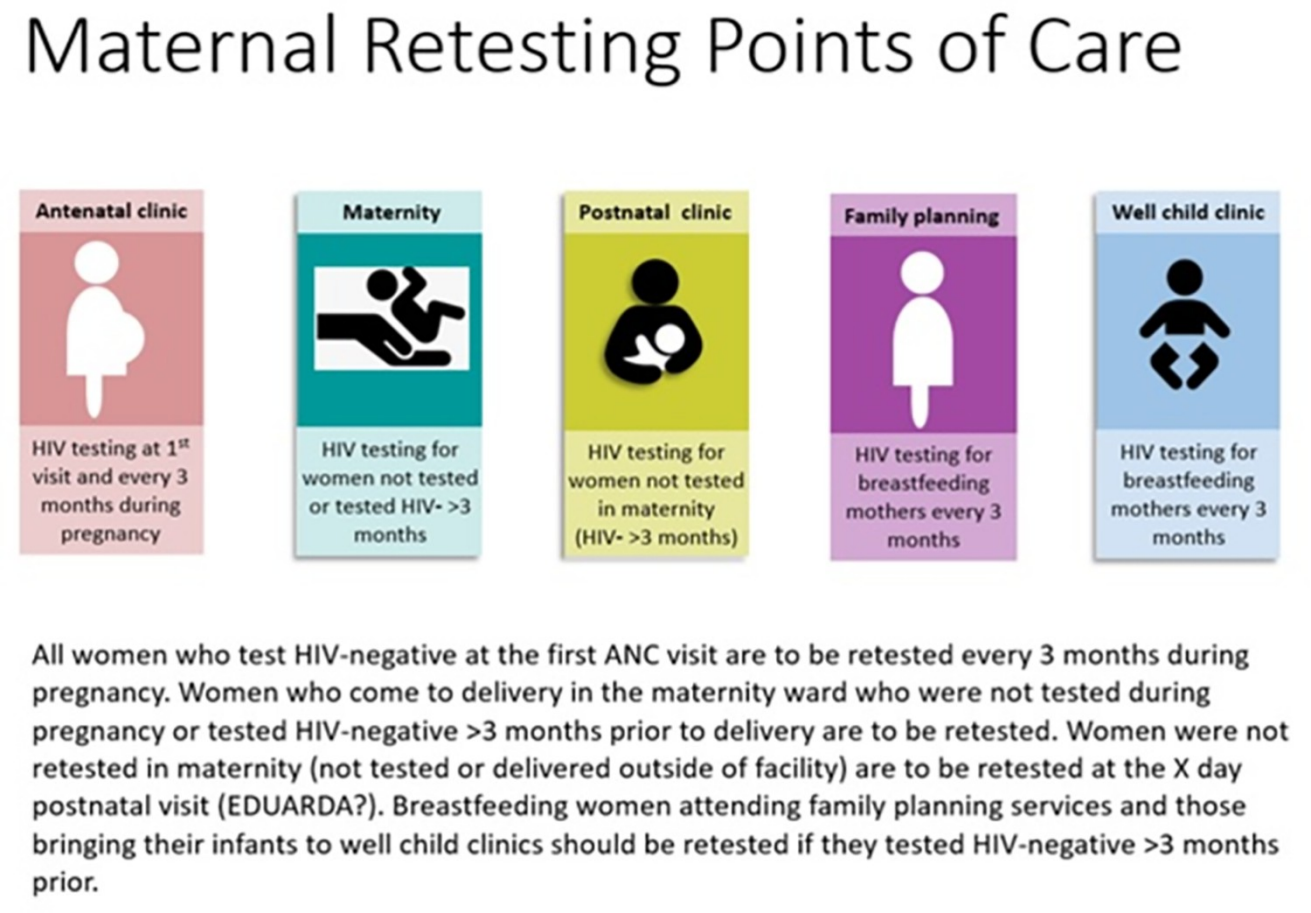
Maternal testing and retesting points of care per Mozambique’s national guidelines (2015) [15].

The project was conducted at 10 health facilities in Nampula province, Mozambique from April through November 2019. The health facilities were purposively selected based on high volumes of women attending both pre- and postnatal care. The aim of the project was to provide support to the facilities to fully implement existing national HIV testing guidelines for pregnant and postnatal women (summarized in Fig 1). Per the guidelines, pregnant women should receive HIV testing at the first ANC visit with retesting every 3 months until cessation of breastfeeding. All pregnant and breastfeeding women coming for care are to be assessed for retesting eligibility (using facility registers or hand-held health cards) in the following settings: ANC, maternity wards (labor and delivery), postnatal clinics (where woman should have postpartum visits at 48 hours, 7, 21 and 28 days), family planning clinics and CWCs. CWCs are clinics within the health facilities where infants and young children from ages four weeks to five years of age are brought to receive weight and growth monitoring checks, immunizations, and nutritional and developmental assessments. Infants and children who are malnourished or sick or have health problems are referred to the at-risk child clinic (CCR) and under-5 clinic consultation (CCD), respectively. All health facilities selected for the project provide all of the services listed.

Project support to the health facilities included monthly data reviews to help improve documentation and ensure eligible women received retesting at all points of care, as well as clinical mentoring for health facility staff focused on identifying and overcoming gaps in testing coverage. Support and clinical mentoring was provided by two trained ICAP nurses and a data manager. Due to high patient volumes in CWCs, one mentor mother (peer educator/mother living with HIV) and two additional trained counselors were hired to screen maternal health cards to identify women eligible for retesting and counselors conducted HIV testing in the CWC at each health facility. Mentor mothers served as patient navigators for women testing HIV-positive. At some facilities, as a result of limited space, it was necessary to erect tents within courtyards to provide private spaces for testing in the CWC. HIV-exposed infants (HEI) (children of women who tested HIV-positive) in CWC were escorted to the CCR for point of care (POC) HIV testing with polymerase chain reaction (PCR). Results were returned to the mother the same day, and children who tested positive were started on ART.

### Data sources and analysis

Data were collected in Mozambican MOH health facility registers by health care providers according to standard procedures. For the analysis, aggregated data reported to the MOH were totaled across the 10 project health facilities for the eight-month project period (data are not shown by facility nor by month). In ANC, data were collected longitudinally on all women who attended a first visit during the evaluation period, including their initial HIV-testing data which was used to assess the number of women eligible for retesting. Among women who tested HIV-negative at the first ANC visit, we report the number who attended a follow-up visit ≥3 months after the first visit (i.e., when eligible for retesting), the number retested at a follow-up visit and retesting results. We present the ANC retesting cascade based on data from women who were eligible for retesting during the project period (April-November 2019), which included women with a first ANC visit starting in January 2019 through August 2019 (women attending a first ANC visit in August 2019 would have been eligible for retesting in November 2019, assuming they were still pregnant). We did not collect gestational age at first ANC visit and did not restrict the analysis to women who came at <6 months pregnant (i.e., those who would have been eligible for retesting during pregnancy).

Outside of the ANC, we were unable to follow women over time to examine retesting however we report aggregated data from the maternity wards, postnatal and family planning clinics which are routinely reported to the MOH and CDC which included the number of women tested and the proportion testing HIV-positive overall and disaggregated by age group. The national registers used in these settings did not allow for differentiation between first and repeat HIV-tests. We consider the women tested in these settings as having been retested based on the assumption that they had been tested previously in ANC. Data reported from family planning clinics included all women tested, some of whom were not postnatal or breastfeeding. We also lack information about the number of unique women eligible for retesting who received care in these settings and thus could not estimate the proportion of women retested among all those eligible.

In the CWC, mothers tested for HIV were also considered to have received repeat testing with the assumption that all had been tested at least once in ANC or at delivery. Maternal retesting data from the CWC were disaggregated by the mother’s age and by the age of the infant when the mother was retested. Similar to the other settings (except ANC), we do not have information about the total number of women eligible for retesting and thus cannot report testing coverage in this venue. The number of women tested and positivity from the CWC were collected throughout the project. Data for the testing cascade, including infant outcomes, were only available for the months July through November (data were missing for April through June) and include the number of women newly testing HIV-positive who were linked to ART (meaning referral rather than confirmation of treatment initiation), the number of children of women who newly tested HIV-positive who had PCR testing for EID, the number who had a positive PCR test and the number of PCR-positive children linked to ART. Reported data include frequencies and proportions which were calculated in SAS 9.4.

The project evaluation was reviewed by the Columbia University Medical Center Institutional Review Board and approved by the Ministry of Health National Committee on Bioethics for Health in Mozambique. The protocol was also reviewed in accordance with Centers for Disease Control and Prevention (CDC) human research protection procedures.

### Results

**Fig 2:**
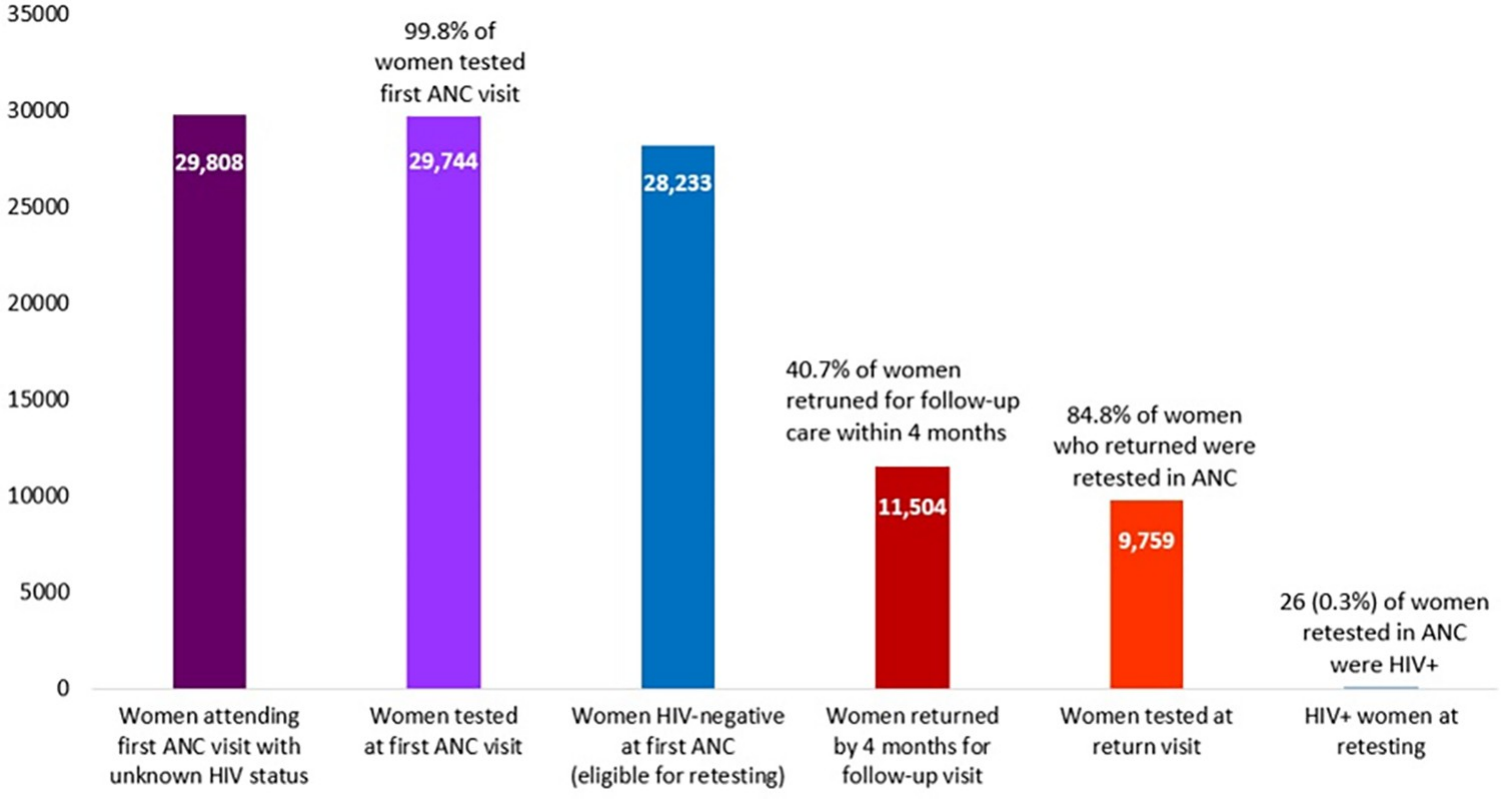
Antenatal clinic HIV retesting cascade, 10 health facilities in Nampula, Mozambique, April-November 2019.

Over eight months in 2019, across 10 health facilities in Nampula, Mozambique, a total of 84,845 HIV tests were provided to pregnant and postnatal women in maternal and child health services, including 55,101 that were likely repeat testing. Data from antenatal clinics show that 29,808 pregnant women with unknown HIV-status attended a first visit from April through November 2019 and 99.8% of those women were tested (Fig 2). Among the 28,233 women who were HIV-negative at the first visit, only 11,504 (40.7%) returned for a follow-up visit >3 months after the first visit and were thus eligible for HIV retesting. Most women (n = 9,759, 84.8%) who returned for a follow-up visit when they were eligible for retesting received testing and 26 (0.3%) tested positive for HIV.

During this time period, 4,468 women were retested in maternity at the time of delivery and 1.2% (n = 52) tested HIV-positive; 697 women were retested in the postnatal clinic and 1.0% (n = 7) tested HIV-positive; 678 were retested in the family planning clinic and 2.1% (n = 14) tested HIV-positive; and 39,499 women were retested in the CWC, where 1.0% (n = 397) tested positive for HIV. Highest test-positivity was observed among young adolescent women 10–14 years: in maternity 10.3% (4 out of 39 tested) and in family planning clinics 16.7% (3 out of 18 tested) tested HIV-positive. Only three women 40 years and older tested HIV-positive in CWC (none tested positive in other settings) (Table 1). Test-positivity was similar for older adolescents (15–19 years) across all settings and was highest in the family planning clinics among 30-34-year-old women (3.6%) (Table 1).

**Table 1:** Women retested in maternity, postnatal, family planning and child welfare clinics and proportion HIV-positive by age, Nampula, Mozambique, April-November 2019.

	All women	Maternal age (years)						
		10–14	15–19	20–24	25–29	30–34	35–39	40+
**Maternity (labor & delivery)**								
Women retested	4468	39	1280	1408	947	530	215	49
Women tested HIV+	52	4	14	17	9	6	2	0
Percent HIV-positive	1.2%	10.3%	1.1%	1.2%	1.0%	1.1%	0.9%	0.0%
**Postnatal clinic**								
Women retested	697	18	221	240	127	50	37	4
Women tested HIV+	7	3	3	0	0	1	0	0
Percent HIV-positive	1.0%	16.7%	1.4%	0.0%	0.0%	2.0%	0.0%	0.0%
**Family planning clinic**								
Women retested	678	1	137	253	196	56	23	12
Women tested HIV+	14	0	2	8	2	2	0	0
Percent HIV-positive	2.1%	0.0%	1.5%	3.2%	1.0%	3.6%	0.0%	0.0%
**Child welfare clinics**								
Women retested	39499	160	11096	13257	7780	4193	2116	897
Women tested HIV+	397	2	82	133	102	52	23	3
Percent HIV-positive	1.0%	1.3%	0.7%	1.0%	1.3%	1.2%	1.1%	0.3%

**Fig 3:**
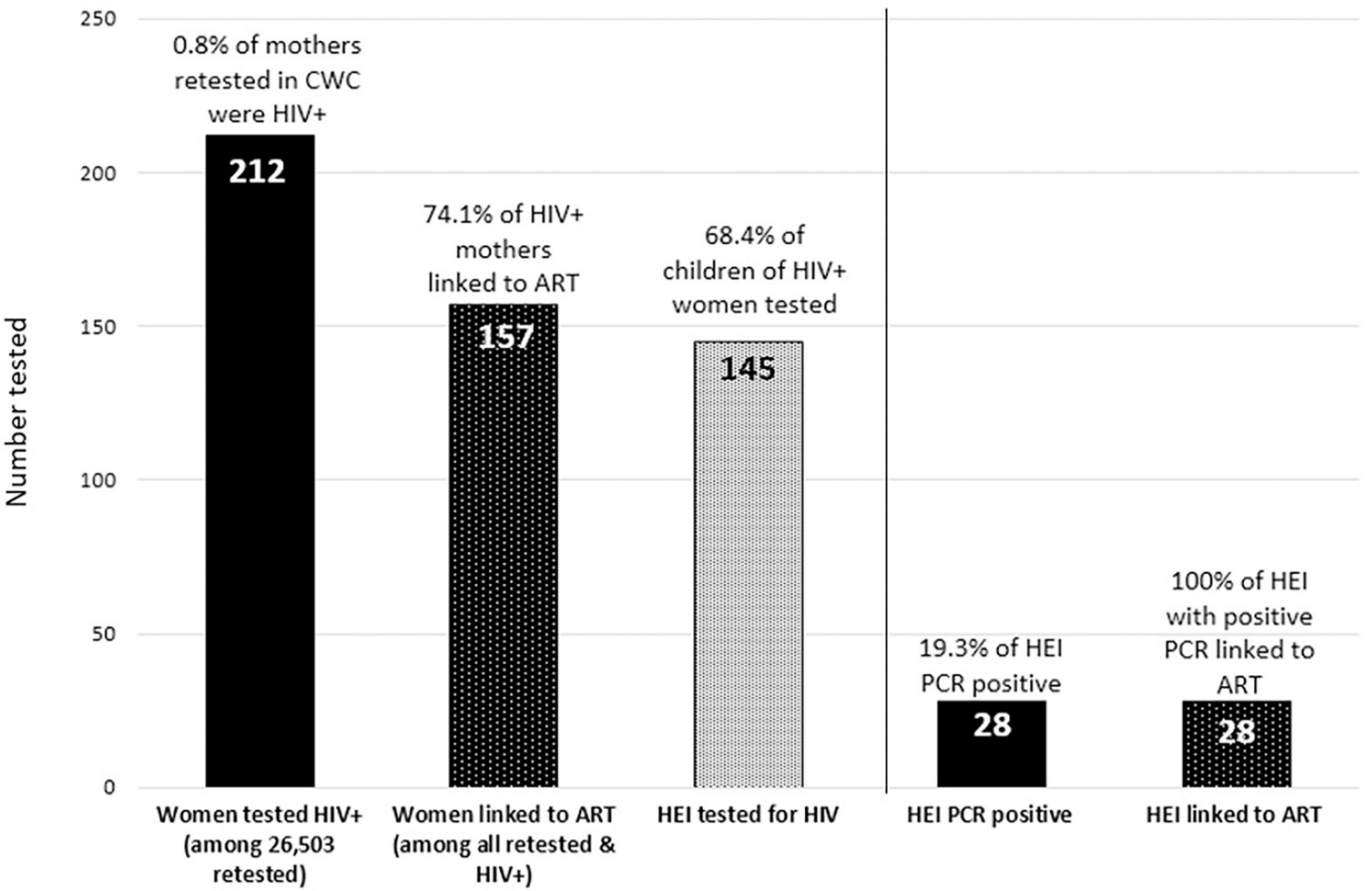
Maternal and HIV-exposed infant testing in child welfare clinics, Mozambique July-November 2019.

**Fig 4:**
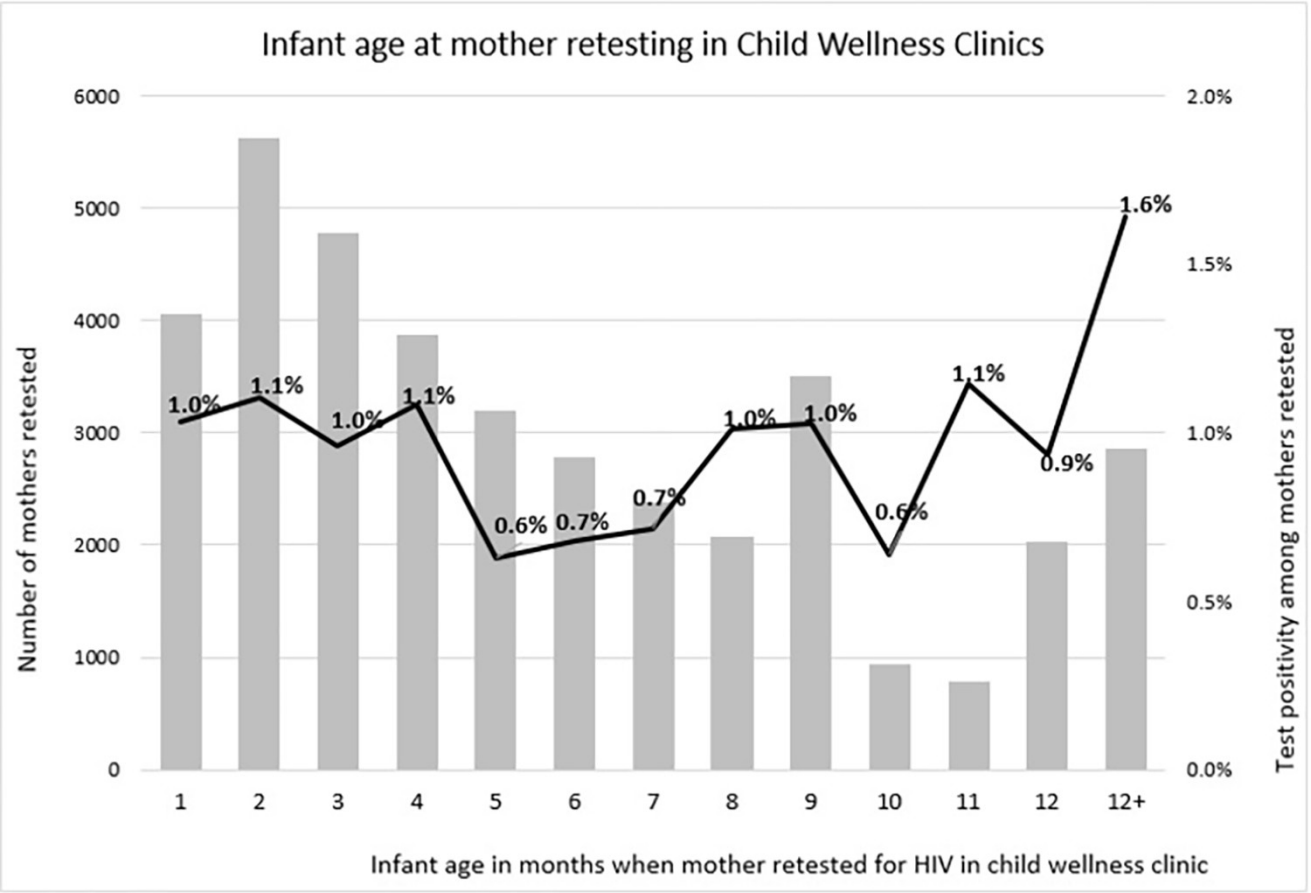
Number of mothers retested for HIV in child welfare clinics and test-positivity among mothers according to age of infant, Nampula, Mozambique, July-November, 2019.

Among 26,503 women retested in the CWC from July through November of 2019, 212 (0.8%) tested HIV-positive and 157 (74.1%) of these women were referred to ART services (Fig 3). One hundred and forty-five (68.4%) infants of mothers who newly tested HIV-positive in the CWC received a PCR test and 19.3% (n = 28) tested PCR-positive. All 28 (100.0%) infants testing PCR-positive were recorded as being referred for ART. In the CWC, mothers of infants 1–3 months of age were the largest group who were retested, whereas few mothers of children who were 10 and 11 months of age received retesting (Fig 4). Maternal test positivity according to the infant’s age was lowest for mothers whose infants were 5 months and 10 months at the time she was retested and was highest among mothers of infants >12 months of age (0.6% and 1.6% respectively).

## Discussion

While many countries have adopted guidelines calling for retesting of pregnant and breastfeeding women, our study is one of the first to provide a perspective on the scale of repeat testing. In addition, our analysis also showed that in ANC, lack of return for antenatal care led to poor HIV retesting coverage in pregnant women. It is noteworthy that almost all women returning for care when eligible received repeat testing. In other service points, we were limited to examination of test yield by age group, which was highest in family planning clinics and among young adolescents (10–14 years) in maternity wards. Finally, our data on HIV testing for mothers in child welfare clinics identified several critical issues, including very large volume of women in need of retesting, poor linkage to testing for HIV-exposed infants, and low test positivity among mothers but high positivity among HEI.

In these ten health facilities, coverage of HIV testing was high at first ANC visit and among those returning for follow-up care during pregnancy. Retesting yield was low with <1% of returning pregnant women determined to have HIV infection. These indicators suggest fidelity to national guidelines in ANC and low rates of incident HIV infections among pregnant women retained in ANC. However, the fact that only 41% of pregnant women returned for a follow-up visit ≥3 months after a first ANC visit is alarming. It is possible that some women went to other clinics after their initial ANC visit and had repeat testing at those facilities [16]. Unfortunately, Mozambique does not have unique health identifiers that would allow for the tracking of women across different health facilities so we were unable to estimate how many women sought follow-up care and received follow-up testing in other facilities. Better information systems that allow for tracking of women across health facilities are needed to measure retesting and to document outcomes of mother and children. Furthermore, some women may have attended their first ANC visit at >6 months’ gestation and therefore did not reach retesting eligibility during pregnancy. However, in a 2019 study conducted in Nampula, 40% of pregnant women attended their first ANC visit by 16 weeks [17] indicating that most women tested at the first ANC visit should have had a least one additional ANC visit prior to delivery where they could have received retesting.

Poor ANC attendance is a significant problem globally [18], however, in high HIV burden settings, there is increased risk to the health of women, who may not start or continue ART, and to their infants. Of particular concern are infants of mothers who acquire HIV during pregnancy and breastfeeding who are at higher risk for vertical transmission and may have delayed ART initiation due to missed diagnosis [7]. Data from UNAIDS showed that in 2020 in the 21 focus countries, 31,000 new pediatric infections resulted from women not remaining in ante- and post-natal care and 27,000 resulted from newly acquired infections during pregnancy or breastfeeding [2]. In order to retest women who initially test HIV-negative in pregnancy, women must remain engaged in care during pregnancy and throughout breastfeeding. Unfortunately, there has been little research on interventions to improve adherence to care among pregnant and breastfeeding women in sub-Saharan Africa, as a recent systematic review reported [19]. While retaining HIV-positive women remains an important focus, effective strategies for improving retention of all pregnant and postnatal women are urgently needed, including efforts to prevent women from acquiring HIV [20]. Key components of enhanced ANC and MCH services for pregnant and postnatal women should include male partner engagement with HIV testing and immediate linkage to ART for anyone positive, as well as expanded access to pre-exposure prophylaxis (PrEP) which is safe for pregnant women and recommended by the WHO to prevent HIV acquisition in mothers and infants [21–23].

Outside of the ANC, our data were limited to test-positivity, however, they provide information that can be used to target services. We found that family planning clinics had the highest testing yield across all age groups, suggesting that the post-partum period is a critical point for repeat HIV testing and that family planning clinics should be targeted to ensure all women are tested and have easy access to PrEP. Although relatively few young adolescents were tested, the highest testing yield in both maternity and family planning clinics was among 10–14 year-olds. Due to the small sample size, we are cautious in interpreting these findings however they are consistent with previous observations that adolescents are less likely to enroll in ANC and often enroll late, making them more likely to miss repeat testing opportunities during pregnancy [24] which may explain why they were identified in maternity and post-natal clinics (rather than ANC). In addition, adolescents are more likely to drop out of services after testing HIV-positive [25]. It is possible that some adolescents at these facilities who tested HIV-positive at delivery and in the postnatal period had previously tested HIV-positive and subsequently dropped out of care. These findings underscore the importance of offering repeat testing for adolescent pregnant and postnatal women across the MCH cascade and the need to offer services targeted to their specific psychosocial needs to improve retention in this vulnerable group [26, 27].

Unfortunately, our findings from child welfare clinics were not encouraging. Among mothers who tested HIV-positive, linkage to testing for infants was poor and almost 20% of those tested were also found to test PCR-positive. We do not have data on whether and when these women previously tested HIV-negative but the high rate of positivity among infants was likely due to the combination of incident infections during pregnancy or breastfeeding, and from women with established infections who did not receive ART during pregnancy. It is very concerning that one third of HEI identified in child welfare clinics were not linked to HIV testing despite efforts to immediately test all HEI on the same day using POC testing. Greater efforts are needed to ensure that all HIV-exposed infants receive diagnostic testing and have a final HIV status determination after the cessation of breastfeeding. These services are critical so that both mothers and infants who are HIV-positive can be initiated on treatment immediately to reduce the risk of morbidity and mortality [28].

Finally, it is important to note the scale of this project as an indicator of magnitude of the retesting mandate for pregnant and postnatal women per Mozambique’s national guidelines. At the 10 project sites, based on facility data, we estimate that each month almost 4,000 pregnant women attended antenatal care services, 3,500 of whom were HIV-negative at the first visit, there were more than 2,700 deliveries and 11,000 children were brought to child welfare clinics. While we do not have data on what proportion of women attending CWC were eligible for retesting, over the eight months, 40,000 women were tested in CWCs across the 10 health facilities (500 women tested per site per month). Fortunately, most mothers attending these services were found to be HIV-negative, however retesting thousands of women each month along the MCH cascade requires staff to conduct tests as well as consistent test kit supplies. It is clear that strategies to target testing to those most at risk and making testing more feasible are urgently needed if repeat testing guidelines are to be fully implemented. Screening tools and other approaches that can effectively identify high risk women are needed. One approach that should be explored is self-testing which has been used effectively in ANC [29, 30] and could be expanded to family planning clinics and other MCH services.

While our data provide important new information about implementation of repeat testing for pregnant and postpartum women in Mozambique, it also has limitations. The lack of data on gestational age at first ANC visit may have led to an overestimate of the proportion of pregnant women who did not return for retesting. While we were able to look at testing and retesting in the same women in the ANC, in other care settings we were unable to do this. A further limitation is that we did not have a denominator for the number of unique women who were tested outside of ANC so we cannot say how many women who were eligible for retesting received it and can also not identify how many unique women were retested (the same women may have been tested multiple times during the project period). In addition, other than in the ANC, we could not distinguish women tested for the first time versus those receiving repeat testing, in particular in family planning clinics. However, given the very high rate of HIV testing at first ANC visit, it is likely that most of the testing that occurred among women in the postpartum period was repeat testing. Finally, the project did not collect process indicators to measure improvement in services prior to and during implementation nor could we compare uptake of testing at these sites to other health facilities.

## Conclusions

Supporting the full rollout of HIV retesting guidelines may be challenging given the high volume of HIV-negative women in need of repeated testing. Innovative strategies and enhancements to the MCH service platform will be needed to ensure the feasibility of full-scale implementation.

## Supporting information

S1 File Inclusivity in global research. (DOCX)

## List of abbreviations

ANC: Antenatal care
ART: Antiretroviral therapy
CWC: Child Welfare Clinics
HEI: HIV-exposed infant
MCH: maternal child health
PMTCT: Prevention of mother-to-child transmission
PRC: polymerase chain reaction
WLHIV: women living with HIV
CDC: US Centers for Disease Control and Prevention
WHO: World Health Organization

## References

[1] Chi BH, Mbori-Ngacha D, Essajee S, Mofenson LM, Tsiouris F, Mahy M, et al. Accelerating progress towards the elimination of mother-to-child transmission of HIV: a narrative review. J Int AIDS Soc. 2020 Aug;23(8):e25571. doi: 10.1002/jia2.25571 32820609 PMC7440973

[2] UNAIDS. Start free, stay free, AIDS free: Final report on 2020 targets. Geneva, Switzerland; 2021.

[3] de Beer S, Kalk E, Kroon M, Boulle A, Osler M, Euvrard J, et al. A longitudinal analysis of the completeness of maternal HIV testing, including repeat testing in Cape Town, South Africa. J Int AIDS Soc. 2020 Jan;23(1):e25441. doi: 10.1002/jia2.25441 31997583 PMC6989397

[4] Woldesenbet S, Jackson D, Lombard C, Dinh TH, Puren A, Sherman G, et al. Missed Opportunities along the Prevention of Mother-to-Child Transmission Services Cascade in South Africa: Uptake, Determinants, and Attributable Risk (the SAPMTCTE). PLoS One. 2015;10(7):e0132425. doi: 10.1371/journal.pone.0132425 26147598 PMC4492960

[5] Knettel BA, Cichowitz C, Ngocho JS, Knippler ET, Chumba LN, Mmbaga BT, et al. Retention in HIV Care During Pregnancy and the Postpartum Period in the Option B+ Era: Systematic Review and Meta-Analysis of Studies in Africa. J Acquir Immune Defic Syndr. 2018;77(5):427–38. doi: 10.1097/QAI.0000000000001616 29287029 PMC5844830

[6] Dinh TH, Delaney KP, Goga A, Jackson D, Lombard C, Woldesenbet S, et al. Impact of Maternal HIV Seroconversion during Pregnancy on Early Mother to Child Transmission of HIV (MTCT) Measured at 4–8 Weeks Postpartum in South Africa 2011–2012: A National Population-Based Evaluation. PLoS One. 2015;10(5):e0125525. doi: 10.1371/journal.pone.0125525 25942423 PMC4420458

[7] Drake AL, Wagner A, Richardson B, John-Stewart G. Incident HIV during pregnancy and postpartum and risk of mother-to-child HIV transmission: a systematic review and meta-analysis. PLoS Med. 2014 Feb;11(2):e1001608. doi: 10.1371/journal.pmed.1001608 24586123 PMC3934828

[8] UNAIDS. Start Free Stay Free AIDS Free: 2018 Progress Report. Geneva, Switzerland: UNAIDS; 2018.

[9] WHO. Consolidated guidelines on HIV testing services. Geneva, Switzerland; 2015.

[10] Drake AL, Thomson KA, Quinn C, Newman Owiredu M, Nuwagira IB, Chitembo L, et al. Retest and treat: a review of national HIV retesting guidelines to inform elimination of mother-to-child HIV transmission (EMTCT) efforts. J Int AIDS Soc. 2019 Apr;22(4):e25271. doi: 10.1002/jia2.25271 30958644 PMC6452920

[11] Jamieson D, Kellerman SE. The 90 90 90 strategy to end the HIV Pandemic by 2030: Can the supply chain handle it? J Int AIDS Soc. 2016;19(1):20917. doi: 10.7448/IAS.19.1.20917 27370169 PMC4930545

[12] Rogers AJ, Weke E, Kwena Z, Bukusi EA, Oyaro P, Cohen CR, et al. Implementation of repeat HIV testing during pregnancy in Kenya: a qualitative study. BMC Pregnancy Childbirth. 2016;16(1):151. doi: 10.1186/s12884-016-0936-6 27401819 PMC4940827

[13] Rogers AJ, Akama E, Weke E, Blackburn J, Owino G, Bukusi EA, et al. Implementation of repeat HIV testing during pregnancy in southwestern Kenya: progress and missed opportunities. J Int AIDS Soc [Internet]. 2017 Dec;20(4). Available from: https://www.ncbi.nlm.nih.gov/pubmed/29236362 doi: 10.1002/jia2.25036 29236362 PMC5810348

[14] Nungu SI, Mghamba JM, Rumisha SF, Semali IA. Uptake and determinants for HIV postpartum re-testing among mothers with prenatal negative status in Njombe region, Tanzania. BMC Infect Dis. 2019;19(1):398. doi: 10.1186/s12879-019-4062-8 31072332 PMC6506942

[15] (misau) M da S. Directriz Nactional Para a Implementacao do Aconselhamento e Testagem em Saude. Maputo, Mozambique; 2015.

[16] Clouse K, Vermund SH, Maskew M, Lurie MN, MacLeod W, Malete G, et al. Mobility and Clinic Switching Among Postpartum Women Considered Lost to HIV Care in South Africa. J Acquir Immune Defic Syndr. 2017;74(4):383–9. doi: 10.1097/QAI.0000000000001284 28225717 PMC5324708

[17] Reis-Muleva B, Duarte LS, Silva CM, Gouveia LMR, Borges ALV. Antenatal care in Mozambique: Number of visits and gestational age at the beginning of antenatal care. Rev Lat Am Enfermagem. 2021;29:e3481. doi: 10.1590/1518-8345.4964.3481 34730761 PMC8570256

[18] Moller AB, Petzold M, Chou D, Say L. Early antenatal care visit: a systematic analysis of regional and global levels and trends of coverage from 1990 to 2013. Lancet Glob Health. 2017 Oct;5(10):e977–83. doi: 10.1016/S2214-109X(17)30325-X 28911763 PMC5603717

[19] Esopo K, Derby L, Haushofer J. Interventions to improve adherence to antenatal and postnatal care regimens among pregnant women in sub-Saharan Africa: a systematic review. BMC Pregnancy Childbirth. 2020;20(1):316. doi: 10.1186/s12884-020-02992-y 32448165 PMC7245828

[20] Elmusharaf K, Byrne E, O’Donovan D. Strategies to increase demand for maternal health services in resource-limited settings: challenges to be addressed. BMC Public Health. 2015;15:870. doi: 10.1186/s12889-015-2222-3 26350731 PMC4562346

[21] WHO. WHO Technial Brief: Preventing HIV during pregnancy and breastfeeding in the context of PREP [Internet]. Geneva, Switzerland: WHO; 2017. Available from: https://apps.who.int/iris/bitstream/handle/10665/255866/WHO-HIV-2017.09-eng.pdf

[22] Joseph Davey DL, Bekker LG, Gomba Y, Coates T, Myer L, Johnson LF. Modelling the potential impact of providing preexposure prophylaxis in pregnant and breastfeeding women in South Africa. AIDS. 2019;33(8):1391–5. doi: 10.1097/QAD.0000000000002221 30950882 PMC6561341

[23] Rosenberg NE, Mtande TK, Saidi F, Stanley C, Jere E, Paile L, et al. Recruiting male partners for couple HIV testing and counselling in Malawi’s option B+ programme: an unblinded randomised controlled trial. Lancet HIV. 2015 Nov;2(11):e483–91. doi: 10.1016/S2352-3018(15)00182-4 26520928 PMC4656790

[24] Mekonnen T, Dune T, Perz J. Maternal health service utilisation of adolescent women in sub-Saharan Africa: a systematic scoping review. BMC Pregnancy Childbirth. 2019;19(1):366. doi: 10.1186/s12884-019-2501-6 31638927 PMC6805384

[25] Kiwanuka J, Mukulu Waila J, Muhindo Kahungu M, Kitonsa J, Kiwanuka N. Determinants of loss to follow-up among HIV positive patients receiving antiretroviral therapy in a test and treat setting: A retrospective cohort study in Masaka, Uganda. PLoS One. 2020;15(4):e0217606. doi: 10.1371/journal.pone.0217606 32255796 PMC7138304

[26] Brittain K, Teasdale CA, Ngeno B, Odondi J, Ochanda B, Brown K, et al. Improving retention in antenatal and postnatal care: a systematic review of evidence to inform strategies for adolescents and young women living with HIV. J Int AIDS Soc. 2021 Aug;24(8):e25770. doi: 10.1002/jia2.25770 34449121 PMC8395389

[27] Teasdale CA, Odondi J, Kidiga C, Choy M, Fayorsey R, Ngeno B, et al. Group antenatal care for improving retention of adolescent and young pregnant women living with HIV in Kenya. BMC Pregnancy Childbirth. 2022 Mar 15;22(1):208. doi: 10.1186/s12884-022-04527-z 35291978 PMC8925235

[28] Violari A, Cotton MF, Gibb DM, Babiker AG, Steyn J, Madhi SA, et al. Early antiretroviral therapy and mortality among HIV-infected infants. N Engl J Med. 2008;359(21):2233–44. doi: 10.1056/NEJMoa0800971 19020325 PMC2950021

[29] Oyaro P, Kwena Z, Bukusi EA, Baeten JM. Is HIV Self-Testing a Strategy to Increase Repeat Testing Among Pregnant and Postpartum Women? A Pilot Mixed Methods Study. J Acquir Immune Defic Syndr. 2020;84(4):365–71. doi: 10.1097/QAI.0000000000002347 32195747 PMC7321872

[30] Sarkar A, Mburu G, Shivkumar PV, Sharma P, Campbell F, Behera J, et al. Feasibility of supervised self-testing using an oral fluid-based HIV rapid testing method: a cross-sectional, mixed method study among pregnant women in rural India. J Int AIDS Soc. 2016;19(1):20993. doi: 10.7448/IAS.19.1.20993 27630096 PMC5023853

