# Correction: HIV retesting for pregnant and breastfeeding women across maternal child health services in Nampula, Mozambique

**DOI:** 10.1371/journal.pone.0315047

**Published:** 2024-12-03

**Authors:** Chloe A. Teasdale, Michelle Choy, Fatima Tsiouris, Eduarda Pimentel De Gusmao, Etelvino C. P. Banqueiro, Aleny Couto, Kwalila Tibana, Nicole Flowers, Marilena Urso, Mirriah Vitale, Elaine J. Abrams

In Fig 1, there is an error in the description indicated in the image. Please see the correct Fig 1 here.

**Fig 1:**
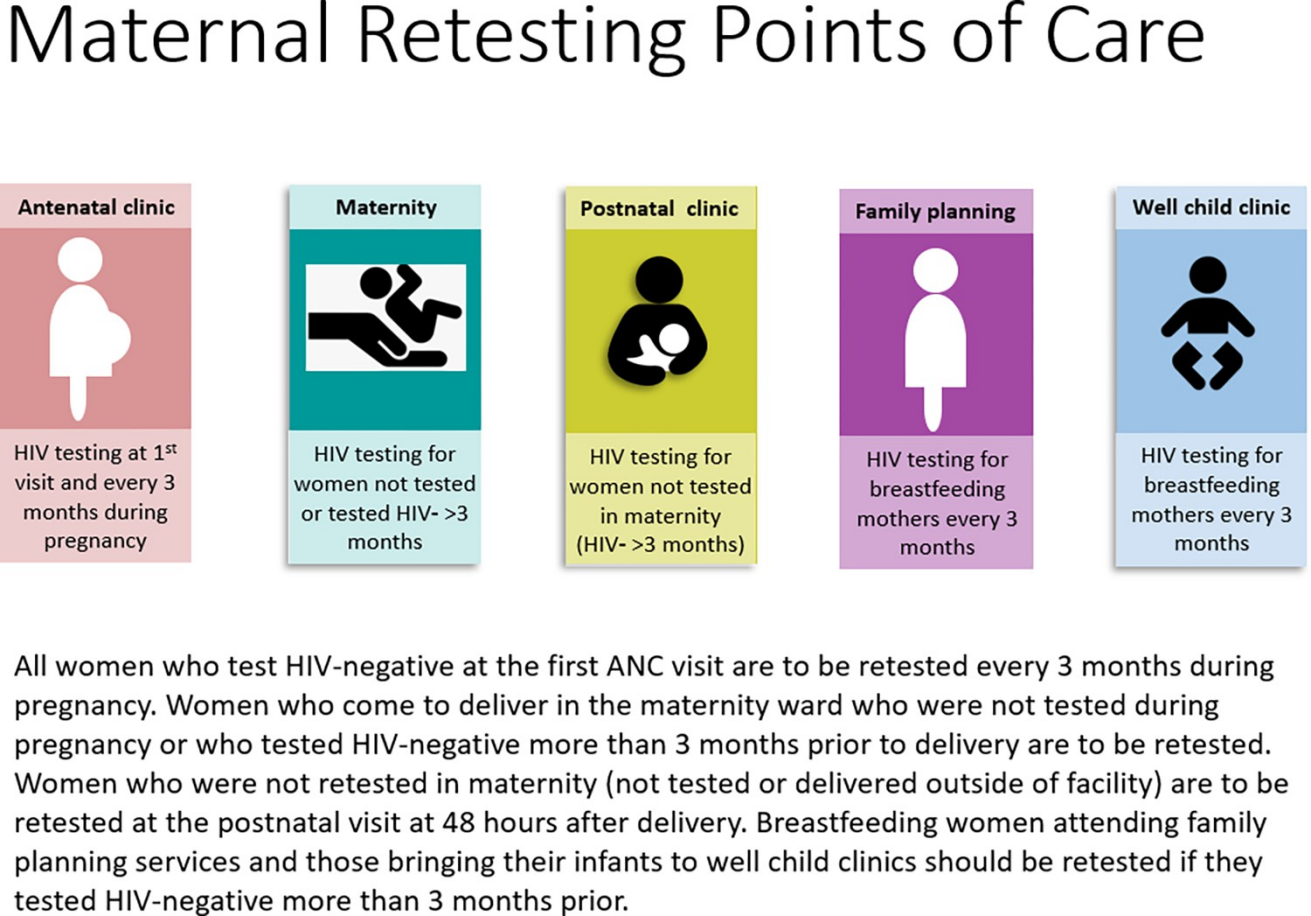
Maternal testing and retesting points of care per Mozambique’s national guidelines (2015) [15].
